# Osmotically Driven and Detected DNA Translocations

**DOI:** 10.1038/s41598-019-51049-4

**Published:** 2019-10-21

**Authors:** Angus McMullen, George Araujo, Michele Winter, Derek Stein

**Affiliations:** Physics Department, Brown University, Providence, Rhode Island 02912 USA

**Keywords:** Nanopores, Physics

## Abstract

A salinity gradient propels a DNA molecule through a solid-state nanopore and generates an ionic current whose change allows for the detection of the translocation. Measurements and theoretical analyses reveal the role of diffusio-osmosis in driving these phenomena: After accounting for known salinity-dependent electrode effects, the measured current change caused by the presence of a DNA molecule inside the nanopore and the DNA translocation speed through it both increase with the magnitude of the applied salinity gradients. The effects are consistent with the theory of diffuisio-osmosis and strong enough to enable DNA translocations to overcome an applied retarding potential of tens of millivolts. This work illustrates how salinity gradients can be used to power and operate a nanopore sensor.

## Introduction

Salinity gradients drive vital transport processes in the membranes of living cells^1^ and play a central role in technological applications like water desalination^2^ and osmotic energy harvesting^3,4^. A salinity gradient holds an entropic form of energy, and the canonical way to harness it is to use a semipermeable membrane^5^. In 1947 Derjaguin described a different mechanism by which salinity gradients could drive transport without a semipermeable membrane^6^: The electrostatic interaction between a charged surface and salt ions causes counterions to accumulate and co-ions to deplete near the interface, and this gives rise to an osmotic pressure gradient that drives fluid flow along the surface. A family of interfacial transport phenomena, which includes diffusio-osmosis, diffusio-phoresis, and chemiosmosis^7,8^ harness osmotic energy and drive transport in this way. They have been used to speed mixing in microfluidic channels^9^ and to convert osmotic energy to electrical power in charged nanotubes^3^.

Osmotic transport is particularly relevant to nanopore biosensors, although this is not widely appreciated at present. A nanopore is a nanometer-scale hole that connects two reservoirs of saline solution and allows an ionic current *I* to flow through it^10^. A nanopore serves as a DNA detector because the presence of a single DNA molecule inside the nanopore causes a measurable change in *I*^11^. Nanopores have garnered considerable interest for their potential applications to genetic analysis and as a unique platform for studying single-molecule biophysics^12^. An applied voltage usually drives the ionic current as well as the passage of DNA molecules through the nanopore^13–16^. Wanunu *et al*. studied nanopore translocations in the presence of salinity gradients, but only considered the influence of the salt concentration on the local electrical conductivity^17^. Sha *et al*. investigated the use of salinity gradients for improving the signal-to-noise of translocation measurements^18^.

Hatlo *et al*.^19^ pointed out that osmotic effects can drive fluid transport in experiments like those of Wanunu *et al*.^17^. But in their theoretical model, Hatlo *et al*. assumed that the nanopore surface is neutral, that its interaction with the solute is repulsive, and that there is a nonzero slip length in the fluid flow profile^19^. Those assumptions are at odds with the fact that the charge densities on the surfaces of silicon nitride nanopores can be highly negative, hence they *attract* counterions^20^, and that it is necessary for the nanopore to be hydrophillic to measure DNA translocations. The model of Hatlo *et al*. predicts diffusio-phoresis entrains DNA toward the lower salinity compartment, propelled by fluid being pumped in the opposite direction^19^, and that prediction is again at odds with the theoretical^6,7,21^ and experimental^9,22,23^ finding that charged channels drive fluid toward the dilute side.

**Figure 1:**
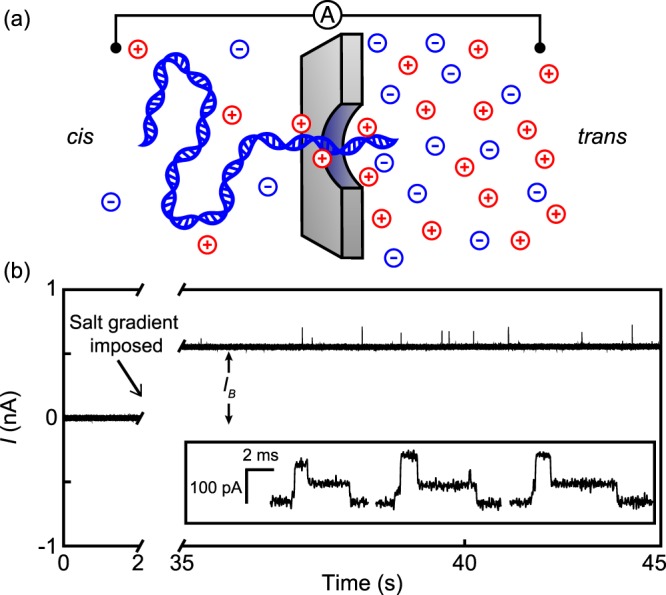
(**a**) Illustration of DNA translocating a nanopore by diffusio-phoresis. DNA moves from the *cis* side, where the salinity is lower, to the *trans* side, where it is higher. (**b**) Ionic current trace from a typical experiment with a 12 nm diameter nanopore. Initially, the solutions in both *cis* and *trans* reservoirs contained 50 mM KCl. Between 2 s and 35 s, the *trans* reservoir was flushed and filled with a 250 mM KCl solution. The resulting shift in *I*_*B*_ is indicated. Inset: selected DNA translocation events observed after imposing the salinity gradient.

To expose and elucidate the transport mechanisms driven by salinity gradients, we used them to power a single-molecule nanopore biosensor. DNA translocations and ionic currents can be driven by salinity gradients alone–i.e. with no applied voltage, as illustrated in Fig. 1a. Our measurements and analysis show that diffusio-phoresis drives DNA through a nanoscale pore up the salinity gradient, while diffusio-osmosis generates an ionic current through the nanopore which rises when the DNA molecule is present inside. These osmotic effects are strong enough to drive DNA through a nanopore against the influence of an applied voltage bias of tens of millivolts, and they are potentially useful in nanopore sensing applications.

## Results

A nanopore-containing chip was placed between two reservoirs, called *cis* and *trans*, each containing a buffered salt solution. Ag/AgCl electrodes were inserted into the reservoirs and connected to a current amplifier (Axon Axopatch) that sensed the ionic current *I* flowing through the nanopore while imposing an arbitrary voltage bias Δ*V* to the *trans* electrode relative to *cis* one. Our fabrication and electrical measurement procedures are described in the Methods and elsewhere^24,25^. Each reservoir had an inlet and an outlet port through which we could flush different solutions without removing the chip or disconnecting the electrodes. We could thereby impose salinity gradients across the nanopore chip, introduce DNA, and control Δ*V* while monitoring *I*. No pressure difference was applied during our measurements.

Figure 1b presents a current trace from an experiment where we imposed a salinity gradient to drive ionic current and DNA molecules through a 12 nm diameter nanopore. We initially filled the *cis* and the *trans* reservoirs with solutions of the same salinity, ${C}_{C}^{s}$ = ${C}_{T}^{s}$ = 50 mM. The *cis* side additionally had about 1 nM of *λ* DNA molecules (48.5 kbp long, New England Biolabs). We zeroed Δ*V* by adjusting the voltage offset of the current amplifier so as to null *I*; under those symmetrical conditions, any electrochemical potential difference that develops across the interface between an electrode and the adjacent solution must be the same in both reservoirs. We next flushed the *trans* reservoir to raise ${C}_{T}^{s}$ to 250 mM. A current of about 0.5 nA began to flow, and we observed transient enhancements in *I* superimposed on that steady current baseline.

The inset of Fig. 1b shows a selection of the ionic current signals. The transient current enhancements are caused by interactions between DNA molecules and the nanopore. The signals in the inset of Fig. 1b are interpretable as translocations by folded DNA molecules, which give rise to the three-level events shown. Those events are remarkably similar to the signals observed in voltage-driven translocation experiments^25^. We used the same custom software to analyze the osmotically driven data as we previously used to study voltage driven nanopore translocations^26^. To focus on the DNA-nanopore interactions, we first subtracted the current baseline *I*_*B*_ from *I* to obtain the current change Δ*I*.

**Figure 2:**
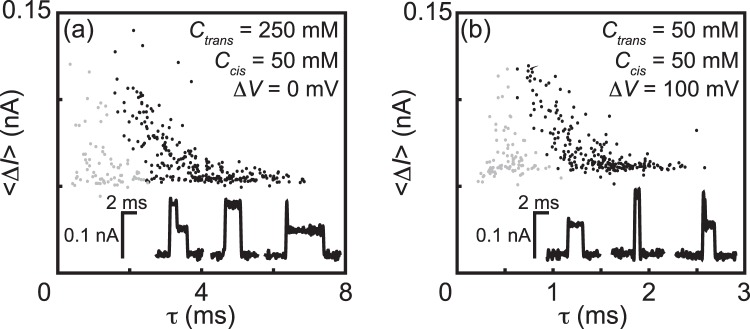
Scatter plots indicating 〈Δ*I*〉 and *τ* of events observed (**a**) with ${C}_{C}^{s}$ = 50 mM, ${C}_{T}^{s}$ = 250 mM and Δ*V* = 0 mV; and (**b**) with ${C}_{C}^{s}$ = ${C}_{T}^{s}$ = 50 mM and Δ*V* = 100 mV. The same 12 nm diameter nanopore was used in both cases. Selected DNA translocation events are shown in (**a**) and (**b**).

Figure 2a is a scatter plot that locates events according to their mean current enhancement amplitude 〈Δ*I*〉 and duration *τ* for an experiment where ${C}_{C}^{s}$ = 50 mM, ${C}_{T}^{s}$ = 250 mM, and Δ*V* = 0 mV were imposed across a 12 nm diameter nanopore. There is a major cluster of events centered at 〈Δ*I*〉 ≈ 0.06 nA and *τ* ≈ 4 ms. There is also a minor cluster near *τ* ≈ 1 ms. For comparison, Fig. 2b shows a scatter plot and a few events from voltage-driven DNA translocations with no salinity gradient (${C}_{C}^{s}$ = ${C}_{T}^{s}$ = 50 mM KCl) and Δ*V* = 100 mV, using the same 12 nm nanopore. There is a major cluster of events centered at 〈Δ*I*〉 ≈ 0.06 nA and *τ* ≈ 1.5 ms, and a minor cluster near *τ* ≈ 0.6 ms. The major clusters of events in both scatter plots correspond to translocations of intact *λ* DNA molecules through the nanopore. We will focus exclusively on those populations of events. The minor populations correspond to brief collisions of DNA with the nanopore that do not result in the molecule’s translocation and to translocations by short *λ* DNA fragments^15,26^. We separated translocations from other events by setting event charge deficit (ECD) thresholds, as previously described^14,25,26^.

**Figure 3:**
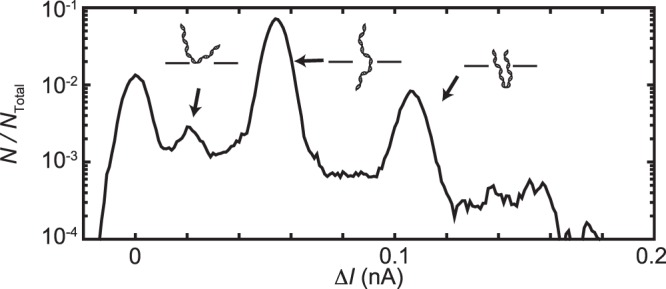
Distribution of 4 *μ*s-long samples of Δ*I*, normalized by *N*_total_, the total number of samples. The ionic current measurements were made with a 12 nm diameter nanopore with ${C}_{C}^{s}$ = 50 mM and ${C}_{T}^{s}$ = 250 mM. The distribution includes data from the translocation events and 100 *μ*s preceding and following each one. Illustrations indicate the physical origin of the various peaks in the distribution.

Figure 3 shows the distribution of 4 *μ*s-long Δ*I* samples from osmotically driven translocations through a 12 nm diameter nanopore. The imposed salinity difference was ${C}_{C}^{s}$ = 50 mM and ${C}_{T}^{s}$ = 250 mM and no voltage was applied. The histogram has four distinct peaks at Δ*I* = 0 nA, 0.020 nA, 0.055 nA, and 0.110 nA. The peak locations were determined by fitting Gaussian distributions to the data, as done in ref.^26^.

Figure 3 corresponds closely to Δ*I* distributions from voltage-driven translocation experiments^11,26,27^, and we assign the same physical interpretation to each peak. In order of increasing Δ*I*, the first peak (Δ*I* = 0 nA) corresponds to the ionic current baseline. The second peak corresponds to a minor, pre-translocation interaction where a molecule docks with the nanopore in a sideways orientation before being pulled through it^28,29^; this sub-state can be seen at the beginning of the translocation events in the inset to Fig. 1b. The third peak corresponds to a single translocating segment of DNA inside the nanopore. The fourth peak, at twice the value of Δ*I* of a single segment, is caused by two DNA segments inside the nanopore at the same time; this occurs when DNA translocates in a folded configuration.

Salinity gradients are clearly capable of driving DNA and ionic current through a nanopore. We now investigate the driving mechanism in more detail. In order to simplify the interpretation of our measurements, we performed the following tests at pH 6, which is near the isoelectric point of silicon nitride^30^. By neutralizing the nanopore surface, we aimed to suppress the diffusio-osmotic effects of the nanopore surface so that we could better focus on the diffusio-osmotic effects at the DNA surface.

**Figure 4:**
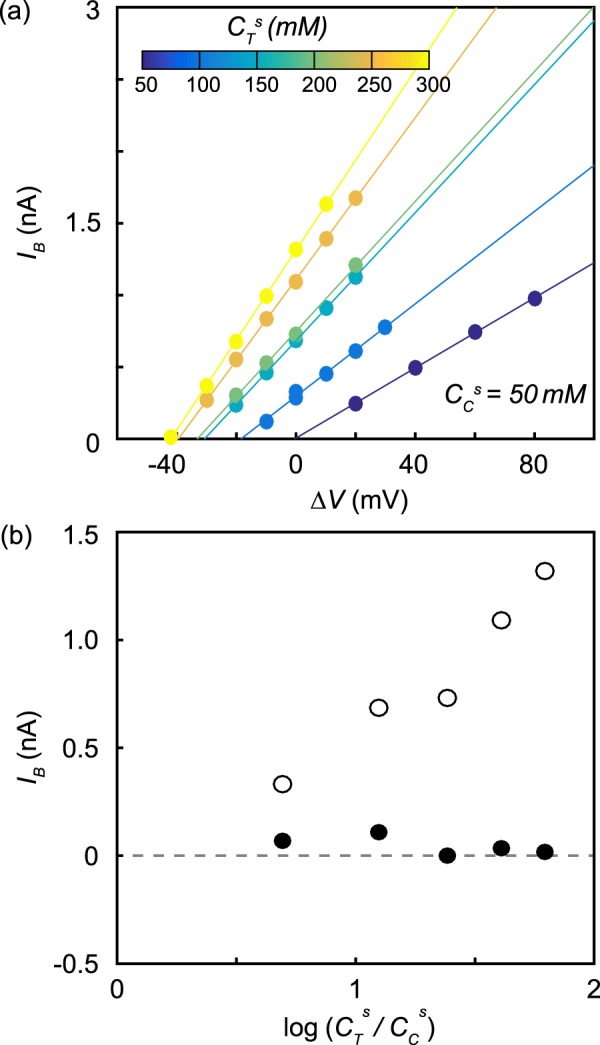
(**a**) Voltage-dependence of *I*_*B*_ for a 22 nm diameter nanopore in six different salinity gradients. ${C}_{C}^{s}$ was held at 50 mM while ${C}_{T}^{s}$ was changed between 50 mM and 300 mM in steps of 50 mM. Error bars were obtained by bootstrap resampling and are the size of the markers when not visible. (**b**) The dependence of *I*_*B*_ (open circles) and *I*_*B*,diff_ (filled circles) as a function of log(${C}_{T}^{s}$/${C}_{C}^{s}$).

Figure 4a shows the dependence of *I*_*B*_ on Δ*V* for six different salinity gradients. ${C}_{C}^{s}$ was held at 50 mM KCl, while ${C}_{T}^{s}$ was varied from 50 mM to 300 mM. These data were measured using the same 22 nm diameter nanopore. We observed and fit a linear relationship between *I*_*B*_ and Δ*V* for all salinity gradients. The slope of that relationship gives the nanopore conductance, *G*_*B*_, which increased as ${C}_{T}^{s}$ increased. The relationship intercepted the voltage axis at Δ*V* = 0 mV when there was no salinity gradient and at increasingly negative values of Δ*V* as the salinity gradient increased. Figure 4b (open circles) shows that *I*_*B*_ measured with no applied voltage increased linearly with log(${C}_{T}^{s}$/${C}_{C}^{s}$).

A salinity gradient is known to cause an electrochemical potential difference to develop between electrodes. We must subtract the effect of that electrode potential from the nanopore conductance to see only the current from diffusio-osmosis^31^

1 $${I}_{B,{\rm{diff}}}={I}_{B}-{G}_{B}\frac{{k}_{B}T}{e}\,\log (\frac{{C}_{T}^{s}{\gamma }_{T}}{{C}_{C}^{s}{\gamma }_{C}}),$$

where *k*_*B*_*T* is the thermal energy, −*e* is the charge of the electron, and *γ* is the salt activity coefficient at the specified concentration^32^. Figure 4b plots the dependence of *I*_*B*,diff_ on log(${C}_{T}^{s}$/${C}_{C}^{s}$). Effectively no baseline current remains that is attributable to diffusio-osmosis after subtracting the current driven by the electrode potential.

**Figure 5:**
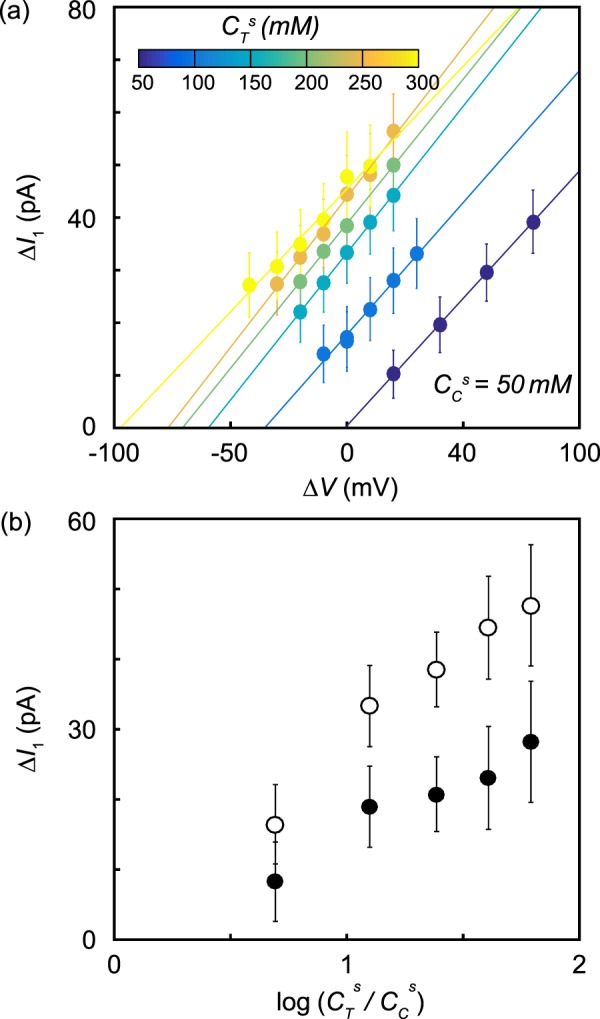
(**a**) Voltage-dependence of Δ*I*_1_ for *λ* DNA translocations through a 22 nm diameter nanopore in six different salinity gradients. ${C}_{C}^{s}$ was held at 50 mM while ${C}_{T}^{s}$ was changed between 50 mM and 300 mM in steps of 50 mM. Error bars were obtained by bootstrap resampling and are the size of the markers when not visible. (**b**) The dependence of Δ*I*_1_ (open circles) and Δ*I*_1,diff_ (filled circles) as a function of log(${C}_{T}^{s}$/${C}_{C}^{s}$).

Figure 5a shows the voltage dependence of the average current change due to the presence of a single dsDNA segment inside the nanopore, Δ*I*_1_, for six different salinity gradients. Again, ${C}_{C}^{s}$ was held at 50 mM KCl while ${C}_{T}^{s}$ was varied from 50 mM to 300 mM. With no salinity gradient, Δ*I*_1_ increased linearly with Δ*V*, and Δ*I*_1_ trends to zero at Δ*V* = 0. DNA translocations enhance current at *C*^*s*^ = 50 mM KCl because the current increase from the entrained counterions exceeds the current sterically blocked by the DNA^27^. When we imposed a salinity gradient, the current enhancement increased but still varied linearly with voltage with a slope, Δ*G*_1_, that did not vary significantly with the imposed salinity gradient. Δ*I*_1_ extrapolated to zero at values of Δ*V* that became increasingly negative as the salinity gradient increased. Figure 5b (open circles) shows that Δ*I*_1_ increased linearly with log(${C}_{T}^{s}$/${C}_{C}^{s}$) when Δ*V* = 0.

We subtracted the effect of the electrode potential on Δ*I*_1_ by measuring the change in conductance caused by the presence of DNA at each salinity gradient (Δ*G*_1_) and then applying the correction

2 $$\Delta {I}_{1,{\rm{diff}}}=\Delta {I}_{1}-\Delta {G}_{1}\frac{{k}_{B}T}{e}\,\log (\frac{{C}_{T}^{s}{\gamma }_{T}}{{C}_{C}^{s}{\gamma }_{C}}).$$

The diffusio-osmotic signals Δ*I*_1,diff_ are plotted in Fig. 5b as black circles. There remained a significant change in current from the presence of DNA in the nanopore due to diffusio-osmosis, even after correcting for the electrode potential, and Δ*I*_1,diff_ increased linearly with log(${C}_{T}^{s}$/${C}_{C}^{s}$).

**Figure 6:**
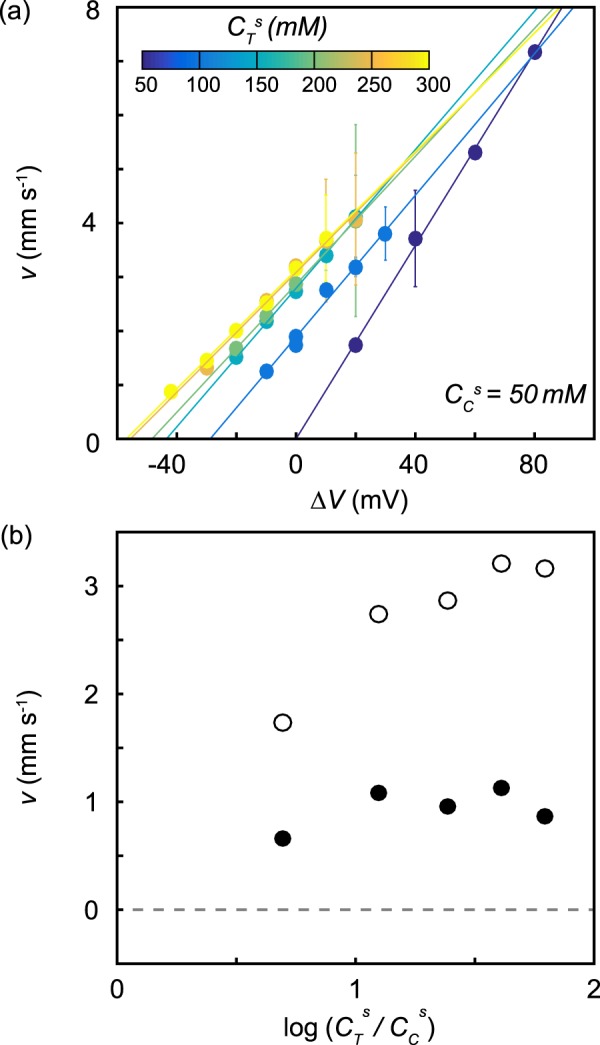
(**a**) Voltage-dependence of *v* for *λ* DNA translocations through a 22 nm diameter nanopore in six different salinity gradients. ${C}_{C}^{s}$ was held at 50 mM while ${C}_{T}^{s}$ was changed between 50 mM and 300 mM in steps of 50 mM. Error bars were obtained by bootstrap resampling and are the size of the markers when not visible. (**b**) The dependence of *v* (open circles) and *v*_diff_ (filled circles) as a function of $\log ({C}_{T}^{s}/{C}_{C}^{s})$.

Figure 6a shows the translocation drift speed, *v*, determined by fitting a first passage time distribution (see Methods) as a function of Δ*V* for the same six salinity gradients and the same 22 nm-diameter nanopore. We see that *v* has a linear dependence on Δ*V* for every condition, and the slope–which corresponds to the voltage-driven translocation mobility, *μ*–decreased modestly as ${C}_{T}^{s}$ increased. The drift speed extrapolated to zero at Δ*V* = 0 mV in the experiment with no salinity gradient and at increasingly negative values of Δ*V* as the salinity gradient increased. Furthermore, we see that the DNA was able to translocate against an applied voltage gradient for every salinity gradient tested. Figure 6b (open circles) shows that *v* tended to increase with log(${C}_{T}^{s}$/${C}_{C}^{s}$) when Δ*V* = 0. The contribution to the drift speed caused by diffusio-phoresis, *v*_diff_, was obtained by subtracting the effect of the electrode potential at each salinity gradient

3 $${v}_{{\rm{diff}}}=v-\mu \frac{{K}_{b}T}{e}\,\log (\frac{{C}_{T}^{s}{\gamma }_{T}}{{C}_{C}^{s}{\gamma }_{C}})\mathrm{.}$$

A significant *v*_diff_ remained after subtracting the effect of the electrode potential.

**Figure 7:**
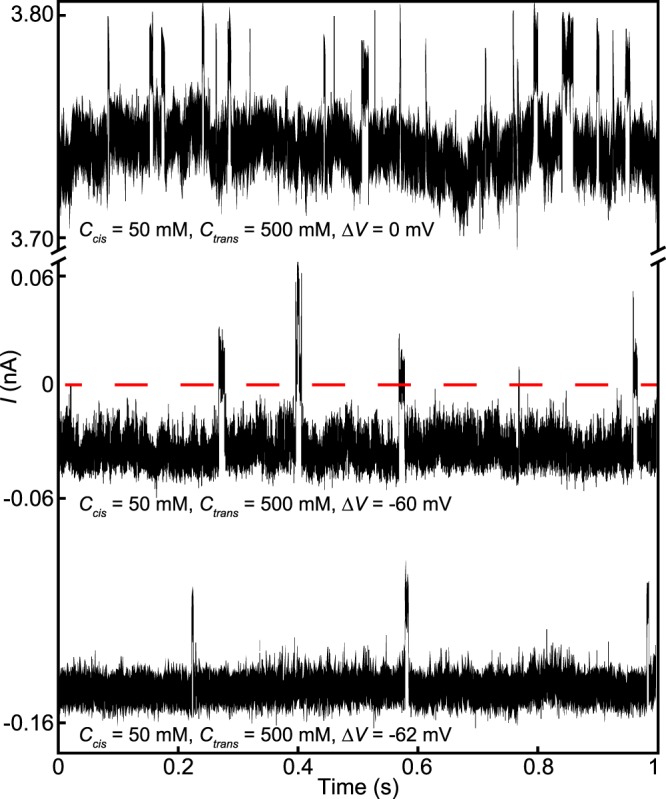
Current versus time traces recorded using a 25 nm diameter nanopore with an imposed salinity gradient of ${C}_{C}^{s}$ = 50 mM and ${C}_{T}^{s}$ = 500 mM and with applied voltages of Δ*V* = 0, −60, and −62 mV, as indicated. Upward deviations from the baseline current are dsDNA translocations.

Figure 7 presents results of an experiment that dramatically illustrates how salinity gradients can drive translocations independently of–and if needed, in opposition to–the electrochemical driving force. Using a 25 nm diameter nanopore, we imposed a salinity gradient of ${C}_{C}^{s}$ = 50 mM and ${C}_{T}^{s}$ = 500 mM and initially set Δ*V* = 0 mV. The resulting current trace shows DNA translocation signals superimposed on a baseline current of *I*_*B*_ = 3.73 nA; the translocations are marked by transient increases in the ionic current. The applied voltage was then set to Δ*V* = −60 mV, which lowered the baseline current to approximately *I*_*B*_ = −30 pA but did not stop the translocation of DNA molecules nor change the direction of the resulting current changes. Finally, the applied voltage was lowered further to Δ*V* = −62 mV, which decreased the baseline current to approximately *I*_*B*_ = −150 pA. Remarkably, DNA translocations were still observed and still caused increases in the total current, even though the sign of the current remained negative throughout. The fact that DNA continued to translocate in the direction opposed to the electrophoretic force clearly demonstrates the importance of diffusio-phoresis in driving translocations. The fact that the current change remained positive while the overall current was negative clearly demonstrates the importance of diffusio-osmosis for detecting translocations.

## Discussion

Diffusio-osmosis and diffusio-phoresis are effects that originate in the force that a charged surface exerts on nearby ions in solution^7,8,33^. The force on an can be expressed ${F}_{q}=-\,q\nabla \Psi$, where Ψ is the electrochemical potential and *q* is the charge of the ion. That force is transmitted to the fluid by the viscous interaction and, at equilibrium, is balanced by a pressure gradient. The force balance in the normal direction is expressed $\frac{\partial p}{\partial z}+e({C}^{+}-{C}^{-})\frac{\partial \Psi }{\partial z}=0$, where *C*^+^ and *C*^−^ are the concentrations of the cations and anions, respectively, *p* is the pressure, and *z* is the normal distance from the surface. In the presence of a slowly varying salinity profile far from the surface, *C*^*s*^(*x*), the local concentration of ions in the double layer is amplified by the Boltzmann factor, $C(x,z)={C}^{s}(x)\,\exp \,(\frac{-q\Psi (z)}{{k}_{B}T})$, leading to a lateral gradient in the osmotic pressure. Here *x* is the lateral coordinate running parallel to the surface. The balance of lateral forces within the double layer gives $\eta \frac{{\partial }^{2}{u}_{x}}{{\partial }^{2}x}-\frac{\partial p}{\partial x}=0$, where *u*_*x*_ is the tangential fluid velocity and *η* is the viscosity. Thus, a salinity gradient generates an osmotic pressure gradient in the double layer, which in turn drives a lateral fluid flow; this is diffusio-osmosis. The diffusio-omotic flow entrains counterions, and this generates an osmotic streaming current that can explain part of the increase in nanopore current when DNA is inside the nanopore. Finally, because a charged object in solution drives a diffusio-osmotic flow down the salinity gradient, it must experience a reaction force pushing it up the salinity gradient; the resulting motion is called diffusio-phoresis and explains the translocation of DNA from *cis* to *trans*.

To make predictions that can be compared with our measurements, we model a section of DNA threaded through the nanopore as a charged cylinder of radius *R*. We also assume that the salinity inside the nanopore varies linearly between ${C}_{C}^{s}$ and ${C}_{T}^{s}$ which are fixed on either side of the membrane whose thickness is *L*. The diffusio-osmotic current that flows along a DNA molecule inside the nanopore is (see Supplementary Information of ref.^3^ for a full derivation)

4 $$\Delta {I}_{1,{\rm{diff}}}=\frac{{k}_{B}T\,R\,\varSigma }{\eta \,{l}_{B}\,L}(1-\frac{{\sinh }^{-1}(\sigma )}{\sigma })\log (\frac{{C}_{T}^{s}}{{C}_{C}^{s}}),$$

where ${l}_{B}={e}^{2}\mathrm{/4}\pi \varepsilon {k}_{B}T$ is the Bjerrum length, *ε* is the permittivity of water, Σ is the surface charge density of the DNA cylinder, and *σ* = 2*πΣλ*_*D*_*l*_*B*_/*e* with *λ*_*D*_ the Debye screening length. Equation 4 applies to planar surfaces; although the radius of DNA is comparable to *λ*_*D*_ in our measurements, we expect it to show only modest deviations from the planar results and no change in the predicted scaling relationships.

Equation 4 predicts a linear relationship between Δ*I*_1,diff_ and log(${C}_{T}^{s}$/${C}_{C}^{s}$), which we observe in Fig. 5b. Using *L* = 20 nm, *R* = 1.1 nm, Σ = 0.85 *e*/nm^2^, and *λ*_*D*_ = 1 nm, Eq. 4 predicts a slope of 22 pA, which agrees reasonably well with the measured slope of 15 pA. The discrepancy could be explained by an effective channel length over which the salinity gradient was established that was longer than *L* = 20 nm.

Turning now to the DNA translocation speed, the diffusio-osmotic fluid velocity parallel to a charged surface reaches^7,21^

5 $${u}_{x}^{\infty }=\frac{{k}_{B}T}{2\pi \eta \,{l}_{B}\,L}\,\log \,\mathrm{(1}-{\alpha }^{2})\log (\frac{{C}_{T}^{s}}{{C}_{C}^{s}})$$

in the far field, where *α* = tanh(*e*Ψ_0_/4*k*_*B*_*T*) with Ψ_0_ the zeta potential of the surface. It is worth mentioning that in addition to being derived for planar surfaces, Eq. 5 neglects a contribution to diffusio-osmosis that originates in a mismatch of the diffusion coefficients of the anions and cations; in the case of potassium chloride, the mismatch is negligibly small. The expression also neglects nonlinear effects that can flip the direction of motion of extremely highly charged objects^7,8,21,33^. Finally, the expression neglects curious entrance effects on diffusio-osmotic transport that theoretical work has recently discovered^34^; such effects are not yet well understood for salt gradients along a charged cylinder like DNA, but we expect them to be of minor importance to the translocation dynamics. Similarly, diffusio-osmotic transport along the inner surface of a nanopore should not depend significantly on its radius. Furthermore, because we took steps to suppress diffusio-osmotic transport along the surfaces of our nanopores, we did not expect, and we did not measure, any significant pore-size dependence.

Equation 5 describes the flows induced by the charged surfaces of both the DNA and the nanopore, with different values of *α* for each surface. The balance of those competing effects determines *v*. As stated previously, we performed our measurements at pH 6 in order to neutralize the nanopore surface (but not the DNA) so that it would not generate any flow by diffusio-osmosis. Therefore, the diffusio-phoretic DNA translocation velocity should be given simply by the negative of Eq. 5, at least for the simple case of a free segment of DNA completely contained within the nanopore. This predicts DNA will translocate in the direction of increasing salinity, as we observed. Equation 5 also predicts *v* should increase linearly with log(${C}_{T}^{s}$/${C}_{C}^{s}$) with a slope of 16 mm/s, assuming Ψ_0_ = −60 mV^35^. A linear fit to the *v*_diff_ data in Fig. 6b gives a slope of 0.7 mm s^−1^, which is about 23 times lower than predicted. But Eq. 5 does not account for the viscous drag forces that act on the parts of the polymer outside the nanopore that the nanopore reels in from large distances^36^, nor does it account for known shortcomings of the nonlinear Poisson-Boltzmann equation^20^. In the case of voltage-driven translocations, those effects suppress *v* by a factor of 14 relative to the analogous theoretical prediction by Ghosal^37,38^. Those effects can explain the discrepancy between the predicted and measured diffusio-phoretic mobilities of DNA in a nanopore in the same way. Overall, the theory of diffusio-phoresis explains *v*_diff_ about as well as the best analytical models of electrophoresis explain voltage-driven translocations.

Diffusio-osmotic phenomena are powerful means of controlling nanopore translocations and signals. They enable salinity gradients to stand in for applied voltages, even though the transport mechanisms are fundamentally different. Remarkably, nanopore sensors can be powered exclusively by a salinity gradient, as demonstrated in Fig. 7, where one sees translocations under the Δ*V* = 0 mV condition. The salinity gradient in that experiment was ${C}_{C}^{s}$ = 50 mM and ${C}_{T}^{s}$ = 500 mM. Under that condition, we ceased to observe translocations when Δ*V* reached about 65 mV, which is close to where *v* extrapolates to zero in Fig. 6 with a similarly high salt gradient of ${C}_{C}^{s}$ = 50 mM and ${C}_{T}^{s}$ = 300 mM. The current changes in Fig. 7 decreased modestly in magnitude with Δ*V*; they are not expected to vanish until Δ*V* reaches a value near −100 mV, according to Fig. 5. We also note that under the Δ*V* = −60 mV condition, with the ionic current close to zero, the external circuit needed to supply almost no power and the electrodes were being consumed at an extremely low rate. The work reported here could lead to the development of salt-powered sensors or sensors that can be operated remotely for extended periods.

## Methods

Nanopores were created in 20 nm thick membranes made of low stress LPCVD silicon nitride using a JEOL 2100 F high-resolution transmission electron microscope (TEM), which was also used to measure the diameter of the nanopore; the detailed fabrication procedure can be found in^24^. Each nanopore was cleaned with a fresh Piranha solution before use. The nanopore was then mounted in a custom made fluidic cell.

The DNA used in all experiments was *λ* DNA, purchased from New England Biolabs. Data shown in Figs 1–3 were collected with pH 8 buffer. Data shown in Figs 4–7 were collected with pH 6 buffer to suppress the charge on the nanopore. Buffers with pH 8 were prepared by first making two stock solutions: one with 1 M KCl, 10 mM Tris, and 1 mM EDTA, and one with simply 10 mM Tris and 1 mM EDTA. Intermediate salt concentrations were prepared by diluting the 1 M stock solution in the KCl-free Tris EDTA buffer. Buffers with pH 6 were prepared with the specified salt concentration and buffered at pH 6 using phosphate buffer.

Ag AgCl electrodes were immersed in both chambers of the fluidic cell and connected to an Axon Axopatch 200B current amplifier, which maintained a constant potential difference while monitoring *I*. The signal was conditioned by an 8-pole Bessel filter with a cutoff frequency of 10 kHz and then digitized using a 250 kHz sampling rate. The data were then filtered with a 10 kHz low pass software filter before data analysis. The fluidic cell was first set up with a symmetric salt concentration of 50 mM KCl and DNA translocations were measured with an applied voltage to test that the nanopore was functioning. The voltage offset on the Axopatch 200B was also set so that the current was ≈0 nA with a symmetric salt concentration and an applied voltage of 0 mV. To impose a salinity gradient, the *cis* side of the fluidic cell was left alone while the *trans* side was flushed with several milliliters of the desired buffer. This was done without turning off the Axopatch or altering the voltage offset. Before imposing a new *trans* salt concentration, we always made sure to correct any drift in the voltage offset by zeroing the current with a symmetric salt concentration.

The analysis of ionic current recordings was performed in two stages using custom MATLAB programs similar to those described in refs^20,26,39,40^. First, a program identified the approximate locations of all significant blockage events in the current recordings. Second, a different program determined the precise times of the start and end of each event, performed an analysis on the current blockage amplitudes over the course of each event, and determined whether the translocation was of a folded or unfolded DNA molecule (from its Δ*I* signature).

We excluded extreme outliers in the integrated current signal of each translocation, also known as event charge deficit (ECD). This is to exclude any DNA fragments (those with very low ECD) or DNA aggregates (those events with very high ECD). This is demonstrated in Fig. 2 by the grey and black points in the scatter plots. To determine the translocation drift speed for each condition, we fit a first passage time function to the distribution of the duration of only unfolded translocations^20^.

## Data Availability

The datasets generated and analysed during the current study are available from the corresponding author on reasonable request.
